# The aging epigenome

**DOI:** 10.7554/eLife.78693

**Published:** 2022-04-28

**Authors:** Brandon L Pierce

**Affiliations:** 1 Departments of Public Health and Human Genetics, University of Chicago Chicago United States; 2 Department of Human Genetics, University of Chicago Chicago United States

**Keywords:** cancer, epigenetic age acceleration, Mendelian randomization, epigenetic clocks, epidemiology, DNA methylation, Human

## Abstract

A new approach helps to assess the impact of accelerated epigenetic aging on the risk of cancer.

**Related research article** Morales Berstein F, McCartney DL, Lu AT, Tsilidis KK, Bouras E, Haycock PC, Burrows K, Phipps AI, Buchanan DD, Cheng I, Martin RM, Davey Smith G, Relton CL, Horvath S, Marioni RE, Richardson TG, Richmond RC, PRACTICAL Consortium. 2022. Assessing the causal role of epigenetic clocks in the development of multiple cancers: A Mendelian randomization study. *eLife* **11**:e75374. doi: 10.7554/eLife.75374.

Age is a prominent risk factor for most types of cancer, including breast, lung and colon cancers, which each have a large impact on public health (de Magalhães, 2013). Cancer risk increases with age, in part, because genetic mutations that arise from DNA replication errors and exposure to environmental carcinogens accumulate as we get older (Tomasetti et al., 2017).

Aging also alters the epigenome, the chemical marks spread across DNA that help switch genes on or off by altering how the genome is packaged. For instance, the addition of a methyl group to DNA can play a role in compressing the nearby DNA sequence so it can no longer be accessed by the cell’s machinery. Epigenetic modifications, including DNA methylation, have also been shown to contribute to the development of cancer (Flavahan et al., 2017; Saghafinia et al., 2018). However, the potential impact of age-related epigenetic changes on cancer development has not been fully characterized.

Previous studies have identified specific DNA methylation sites that are associated with age (Horvath and Raj, 2018). Researchers have developed algorithms, called ‘epigenetic clocks’, that use data from tens to hundreds of these methylation sites to estimate an individual’s ‘epigenetic age’. This includes the Horvath clock which predicts age using DNA methylation data from any tissue type (Horvath, 2013), and the Hannum clock which was designed to use data from blood cells (Hannum et al., 2013).

It has been hypothesized that people whose epigenetic age is greater than their age in years – a phenomenon known as accelerated aging – may be at higher risk of age-related diseases, including cancer (Yu et al., 2020). However, previous studies linking accelerated epigenetic aging and cancer have produced mixed results. Now, in eLife, Fernanda Morales Berstein from the University of Bristol and co-workers (who are based at various institutes in the United Kingdom, the United States, Greece and Australia) report how they tackled this question using a different approach to most prior studies called Mendelian randomization (Morales Berstein et al., 2022). Instead of associating a person’s risk of cancer with epigenetic clock estimates, they correlated it against genetic variations that are known to influence these algorithms.

First, the team examined results from a previously conducted genome-wide association study which had analyzed the DNA of over 34,000 individuals to identify genetic variations that influence epigenetic clocks (McCartney et al., 2021). They used these results to select specific variants that predict the epigenetic age values measured by four common clocks (Horvath, Hannum, PhenoAge and GrimAge).

Next, Morales Berstein et al. used the Mendelian randomization method to find out if the variants that predict accelerated aging also affect the risk of several different types of cancer (breast, prostate, ovarian, colorectal and lung cancer). To do this the team obtained data from several large genome-wide association studies that had searched the genome of individuals for differences that predict cancer status; genetic variations related to the aging clocks were then extracted to see if they were also associated with an increased risk of cancer.

The results of Morales Berstein et al. did not show many clear relationships between the epigenetic aging clocks and risk for the various types of cancer studied. The most promising finding was an association between the GrimAge clock and colorectal cancer. The GrimAge clock was not designed to predict age alone, but also reflects the effects of smoking and other mortality-related epigenetic features (Lu et al., 2019). Thus, the interpretation of this association is not straightforward, as this clock may capture the effects of environmental or lifestyle factors on the epigenome.

While the Morales Berstein et al. study did not show pervasive effects of epigenetic aging on cancer risk, their work is a critical contribution to cancer susceptibility research, as they have addressed an important question using rigorous methods, including Mendelian randomization. The primary strength of studies that use this approach is that they are less prone to certain types of biases that can affect observational research, such as confounding and reverse causation. Furthermore, it is important to acknowledge that epigenetic clocks have largely been developed based on how aging affects DNA methylation in blood cells. Much less is known regarding aging and epigenetics in other tissue types, including those prone to cancer, such as the ones examined by Morales Berstein et al.

Future studies will likely use Mendelian randomization to address similar hypotheses for additional cancer types and a wider variety of epigenetic aging algorithms. As the size of genome-wide association studies increase and more clock-related genetic variants are discovered, this approach will gain more power to detect the effects of epigenetic aging on cancer and other age-related diseases.
